# Bonding Strategies for Thermoplastics Applicable for Bioanalysis and Diagnostics

**DOI:** 10.3390/mi13091503

**Published:** 2022-09-10

**Authors:** Kieu The Loan Trinh, Duc Anh Thai, Nae Yoon Lee

**Affiliations:** 1 Department of Industrial Environmental Engineering, Gachon University, 1342 Seongnam-daero, Sujeong-gu, Seongnam-si 13120, Korea; 2 Department of BioNano Technology, Gachon University, 1342 Seongnam-daero, Sujeong-gu, Seongnam-si 13120, Korea

**Keywords:** thermoplastic polymers, thermoplastic bonding, microfluidic technology, microfabrication, microfluidic device

## Abstract

Microfluidics is a multidisciplinary science that includes physics, chemistry, engineering, and biotechnology. Such microscale systems are receiving growing interest in applications such as analysis, diagnostics, and biomedical research. Thermoplastic polymers have emerged as one of the most attractive materials for microfluidic device fabrication owing to advantages such as being optically transparent, biocompatible, cost-effective, and mass producible. However, thermoplastic bonding is a key challenge for sealing microfluidic devices. Given the wide range of bonding methods, the appropriate bonding approach should be carefully selected depending on the thermoplastic material and functional requirements. In this review, we aim to provide a comprehensive overview of thermoplastic fabricating and bonding approaches, presenting their advantages and disadvantages, to assist in finding suitable microfluidic device bonding methods. In addition, we highlight current applications of thermoplastic microfluidics to analyses and diagnostics and introduce future perspectives on thermoplastic bonding strategies.

## 1. Introduction

Microfluidics is a multidisciplinary technology that is used in various applications including analyses, diagnostics, and biomedical research [1,2,3,4]. Historically, silicon and glass substrates were used for fabricating microfluidic devices, then, the rapid advancement in soft lithography technology allowed using polydimethylsiloxane (PDMS) [5,6]. However, PDMS is limited by its hydrophobic absorption and low mechanical rigidity. Moreover, PDMS device fabrication is relatively complex and has a low throughput [7,8]. Subsequently, thermoplastic materials have been widely applied because of their good mechanical rigidity and high-throughput fabrication [9]. Typical thermoplastic materials for microfluidics include polystyrene (PS), polycarbonate (PC), poly (methyl methacrylate) (PMMA), cyclic olefin copolymer (COC), poly (ethylene terephthalate) (PET), polypropylene, and polyvinyl chloride (PVC). These materials are optically clear, rigid, compatible with many organic solvents, and show low absorption of small molecules [9,10]. These advantages make thermoplastics ideal for analytical microfluidics. However, because these materials are barely permeable to gas, their sealed microchannels are inappropriate for long-term cell culture. Nevertheless, in contrast to traditional materials such as silicon and glass, thermoplastics are recommended for microfluidics due to their low fabrication costs and easy manipulation.

**Figure 1:**
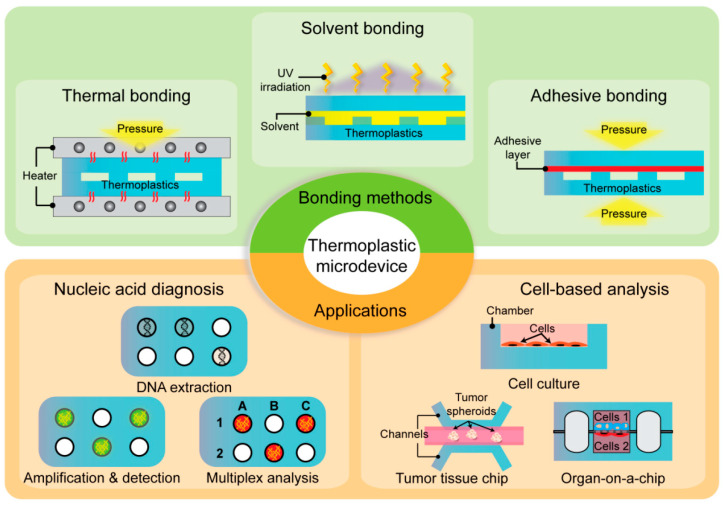
Summary of representative bonding methods (thermal bonding, solvent bonding, and adhesive bonding) and the applications of thermoplastic microfluidics in nucleic acid diagnosis and cell-based analysis.

Apart from material selection and microchannel fabrication, microdevice bonding is a concern in the development of thermoplastic microfluidics. Although thermoplastics share common characteristics, each material possesses unique properties including chemical composition, glass transition temperature, mechanical rigidity, and solvent compatibility [11]. Therefore, there are diverse bonding methods including common strategies such as thermal bonding, solvent bonding, adhesive bonding, and physical/chemical-assisted bonding (Figure 1). Several key factors determine the effective bonding in thermoplastic microdevices. Particularly, bond strength is a critical factor that determines robust and stable bonding without leakage during microdevice operation. Moreover, optical transmittance and biocompatibility should be considered when selecting a bonding method for optical detection and cell research [12]. In this review, we discuss the advantages and limitations of each thermoplastic bonding method. Further, we reviewed multidisciplinary applications of thermoplastic microfluidic devices, such as nucleic acid-based diagnosis, cell manipulation, and organ-on-a-chip (Figure 1).

## 2. Thermoplastic Materials for Microfluidic Fabrications

The fabrication of microfluidic devices with thermoplastics involves a variety of replication methods such as hot embossing, injection molding, imprinting, and thermoforming [13,14,15,16]. Injection molding involves the injection of the molten thermoplastic polymer in a high-precision mold under high pressure. Then, the mold is cooled below the glass transition temperature (T_g_) of the plastic material and the microfluidic structure is formed. This technique enables the fabrication of complex structures, but the fabrication cost is high and it is difficult to assemble separate layers for the construction of closed microfluidic devices [17]. The hot embossing technique is based on pressing a microstructure mold with a force onto a thermoplastic substrate while the substrate is heated slightly above the T_g_. The thermoplastic substrate contains the microstructures after cooling and releasing from the mold. Hot embossing has many advantages, including lower manufacturing costs and easy operation, but parameters such as temperature and force need to be critically controlled to achieve maximum accuracy [18]. Imprinting involves embossing a hard mold into a soft material of thermoplastic polymers to yield small features on large area substrates. A crucial factor to qualify for successful molding is material flowability, since flow resistance can impede the creation of smaller structures [19]. In thermoforming, the thermoplastic sheets are heated and softened, maintaining a solid state (thermoelastic state) without losing material coherence. Thermoforming enables the formation of 3D structures; however, the processing time is relatively long [20]. Recently, thermoplastics have been engraved by direct machining methods including laser ablation and mechanical micromilling [21,22]. Laser ablation relies on an ultraviolet (UV) pulse of laser radiation onto a material to break bonds within a polymeric molecule, which enables the creation of microfluidic structures. Parameters such as wavelength, power, pulse duration, and repetition rate are precisely controlled for the high volume replication of microfluidic devices. A smaller wavelength and beam quality can create finer features. The laser ablation process is realized in a relatively short time, but requires expensive equipment and a high cost for the manufacture [23,24]. Alternatively, micromilling uses a mill with high-speed rotation to create microfluidic structures. Usually, the tool speed and position are automatically controlled by computer numerical control (CNC) programming from a computer-aided design file. CNC milling is used to fabricate prototypes or to create structured molds for the rapid generation of microchips via PDMS casting. Micromilling can easily and rapidly manufacture structures with high aspect ratios and can be an effective and relatively low-cost strategy for prototyping microfluidic devices. Nevertheless, micromilling has the limitations of poor microstructure resolution. The CNC machines may be combined with microscope to monitor the desired sizes and features, improving the accurate tolerances on milled structures [25,26].

Thermoplastics are densely crosslinked polymers that soften when heated to their T_g_ but solidify upon cooling while maintaining their original chemical bonds. Thermoplastics are generally durable due to their chemical and dimensional stability, which makes them highly adaptable for a wide range of microfluidic applications. Depending on the application, both thermoplastic materials and fabrication methods are appropriately selected according to their physical and chemical properties. PS is optically transparent, biocompatible, and inert, making it suitable for cell culture research [27]. The PS surface can be easily functionalized via physical and chemical modifications such as irradiation, gas plasma, and corona discharge to increase surface hydrophilicity [28]. Although PS is an inexpensive material, expensive equipment is required to produce complex chips. Injection molding and hot embossing are commonly used as molding methods for PS [27]. Next, PC is a durable and transparent thermoplastic polymer for microfluidics used in bioanalytic applications such as nucleic acid isolation and pathogen detection. Particularly, PC is appropriate for a range of enzymatic amplifications such as continuous flow polymerase chain reaction (CF-PCR) because its T_g_ is very high (T_g_ = 145–155 °C) [29,30]. However, the fabrication of the PC microstructure depends on hot embossing; thus, the bonding method is limited by thermal bonding since the thermal bonding of PC requires high temperatures which can damage microchannels [31]. PMMA is a cheap and easy-to-fabricate polymer; it is the most common thermoplastic. In addition to its optical transparent and rigid properties, PMMA is biologically compatible with cells and useful for cell research [32,33]. PMMA microfluidic devices are also applied in extraction and electrophoresis separation systems [34]. PMMA patterns can be formed through hot embossing and injection molding. Moreover, this material can easily engrave microchannels by CO_2_ laser or micromachining [9]. COC is an amorphous thermoplastic copolymer made from cyclic monomer polymerization [35]. The COC surface is hydrophobic, causing nonspecific adsorption of analytes. Therefore, its surface chemical modification is necessary. COC is resistant to acids and several organic polar solvents; thus, COC microfluidic systems are attractive for on-chip chromatography [36]. COC exhibits highly optical transparency and low background fluorescence, making it interesting for lab-on-a-chip systems designed for fluorescent detection using integrated circuits [37]. The T_g_ of COC ranges from 70 to 170 °C, depending on polymer content. Moreover, molding methods such as injection molding, compression molding, thermoforming, and many others can be applied to COC materials [38]. Additionally, there are other less common thermoplastics used for microfluidics such as PET and PVC. These materials have a low T_g_ of around 80 °C and good resistance to solvents [39], and both materials can be molded by hot embossing, imprinting, and laser ablation [40].

## 3. Thermoplastic Bonding

### 3.1. Thermal Bonding

**Figure 2:**
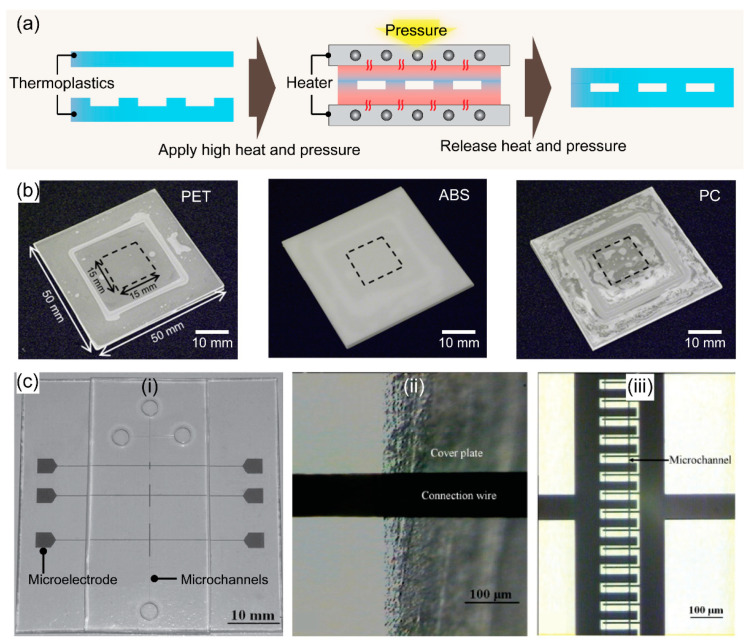
(**a**) Schematics showing the representative procedures for thermal bonding for fabricating thermoplastic devices. (**b**) Photographs of PET, ABS, and PC substrates bonded under the optimal heating conditions at 107, 152, and 202 °C using the thermal bonding method, respectively, the squares indicated by black dotted lines show the parts cut out using the picosecond laser to measure bonding strength. Adapted with permission from Ref. [51]. Copyright 2018, Elsevier. (**c**) PMMA microfluidic device with integrated metal microelectrodes bonded at 85 °C using a plasma-assisted thermal bonding method: (i) photograph of the chip with copper IDMAs, (ii) connection wire at the edge of the cover plate without cracks, (iii) copper IDMAs in the microchannel. Adapted with permission from Ref. [52]. Copyright 2009, Elsevier.

Thermal bonding is a bonding process that uses heat and pressure to seal microfluidic devices. In thermal bonding, two thermoplastic substrates are heated near or above their T_g_, and the substrates become rubbery fusing at the interface under the pressure. This leads to a robust bond between the surfaces due to the crosslinked polymers at the interface, as shown in Figure 2a. Therefore, under optimal bonding conditions, not only similar but also dissimilar thermoplastic substrates are easily sealed using the thermal bonding method. Thus, this bonding method is also called thermal fusion bonding. Thermal bonding is simple and robust, making it the most common method for sealing microfluidic chips [41]. Under ideal conditions, high bond strength can be achieved through a simple process. Moreover, given the direct bonding without intermediate materials, these microchannels present homologous surfaces after bonding which maintain the initial properties of thermoplastic materials [40]. During thermal bonding, the thermoplastic substrates are aligned between supporting plates and a hot press machine applies heat and pressure [42]. Interestingly, the hot press machine can be utilized for both hot embossing microchannel fabrication and thermal bonding. Chen et al. designed a spring-driven press device for hot embossing and thermal bonding of PMMA microfluidic chips. This simple press device consisted of press heads, compression springs, and screw nuts to fix the PMMA plates before heating them in a convection oven for embossing or bonding [43]. Later, the press device had a positive temperature coefficient ceramic heater inside [44].

The T_g_ of the material determines the bonding temperature; therefore, the bonding of thermoplastics with the same or similar T_g_ is recommended to prevent channel deformation. For instance, PMMA bonding was achieved by heating at 90–95 °C for 10 min under a pressure of 1–2 MPa [43,45,46]. In another study, the thermal bonding required heating at over 120 °C for 1 h by a pressure cooker since a specific PMMA with high T_g_ was used [47]. A temperature of 105 °C and a pressure of 0.4 MPa is required to produce low-deformation thermal bonding of PS nanostructured microfluidic chips [48]. Due to the high T_g_ property, PC (T_g_ = 147 °C) and polyimide (PI) (T_g_ > 300 °C) thermal bonding has been performed at 134 °C for 10 min and 380 °C for 5 min, respectively [49,50]. However, thermal bonding poses some drawbacks such as thermal deformation or microchannel collapse during PET, acrylonitrile butadiene styrene (ABS), and PC chip manufacturing (Figure 2b) [51]. In another study, to decrease the bonding temperature, Liu et al. presented plasma-assisted thermal bonding for sealing PMMA microfluidic chips integrated with metal microelectrodes (Figure 2c) [52]. With the use of plasma, the bonding temperature was decreased from 100 to 85 °C due to a lower Tg at the surface of polymers after plasma treatment, and the fracture of copper microelectrodes was eliminated. Adapting to the same concept, Immanuel et al. also introduced surface activation through H_2_O plasma treatment linked with low-temperature annealing for bonding PMMA devices for blood tests [53].

Additionally, thermal bonding can be also applied for sealing hybrid thermoplastic materials such as PMMA–COC and PMMA–TPE (thermoplastic elastomer). The bonding parameters for these materials are usually 70–80 °C for 15 min under a pressure < 1.6 MPa [54,55,56]. Apart from optimizing the bonding parameters (temperature, pressure, and time), surface pretreatments such as gas plasma, UV, and chemical treatments can improve the bonding. Indeed, treating the PMMA substrates with isopropyl alcohol for 75 s in a boiling bath before thermal bonding, improved a four-fold increase in bond strength, with full favorable optical clarity [57]. In addition, a surface treatment can help lower the bonding temperature, reducing the risk of microchannel deformation due to high temperature and force application. The activation of UV/O_3_ light helps PMMA and COC sealing at 70 °C, a temperature significantly below the T_g_ of the substrates [54]. Moreover, plasma-assisted thermal bonding has been shown to be beneficial in increasing bond strength and decreasing bonding temperature [56,58]. 

### 3.2. Solvent Bonding

**Figure 3:**
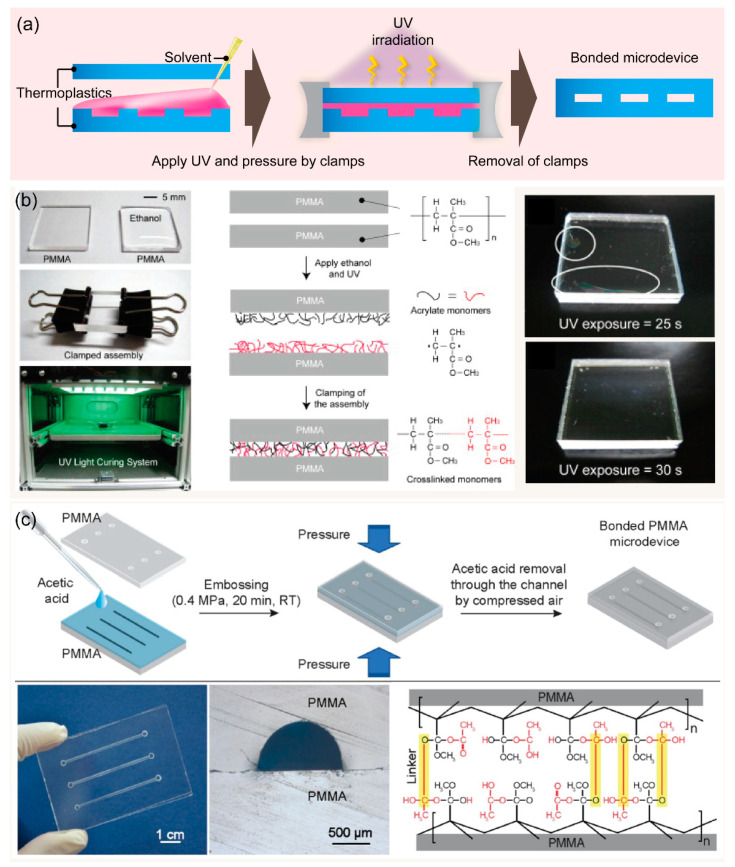
(**a**) Schematics showing the procedures for solvent bonding for fabricating thermoplastic devices. (**b**) The overall procedure for bonding two PMMA substrates via ethanol treatment followed by UV irradiation, a chemical reaction is anticipated to take place on the surfaces of two PMMAs substrates after ethanol and UV treatment. Adapted with permission from Ref. [61]. Copyright 2013, Elsevier. (**c**) The overall procedure for bonding two PMMA substrates at room temperature by acetic acid under pressure. The photographs show bonded PMMA microdevice and cross-section of the microchannels after the bonding, chemical bonds are anticipated to form between two PMMA substrates after acetic acid and pressure treatment. Adapted with permission from Ref. [33]. Copyright 2019, Elsevier.

Solvent bonding is a versatile process commonly used for the permanent joining of thermoplastic materials. In solvent bonding, a solvent is applied to dissolve and break down polymer chains at the contact surface, and then the polymer chains of two substrates are crosslinked to create a permanent bond. After the solvent evaporates, a strong thermoplastic-to-thermoplastic bond is formed even at low temperatures with less requirement of equipment (Figure 3a). Solvent bonding enables robust bonding at a relatively low temperature. The process is fast and inexpensive. Notably, high bond strength of 11.75 and 14.95 MPa can be achieved when applying acetic acid for microwave-assisted or UV-assisted solvent bonding of PMMA microdevices, respectively [59,60]. These bonding strengths are higher than the limits of typical thermal and adhesive bonding. Different types of solvents can be applied for thermoplastic bonding depending on thermoplastic materials. For example, PMMA devices can be bonded using ethanol [61,62], chloroform [63], isopropyl alcohol [64], and acetic acid [36,59,60], while cyclic olefin polymer (COP) sealing can be performed using cyclohexane and toluene [65]. 

Lukashenko et al. investigated a chemical solvent bonding technique for manufacturing nondetachable PMMA substrates using different solvents such as ethyl acrylate, n-butylacrylate, and vinyl acetate. In particular, vinyl acetate was selected since it exhibited the solvent-bonded seam with smaller change in the working volume of microstructures after bonding [66]. An optimized solvent composition for bonding is a key for good solvent bonding performance. For instance, a weak solvent or one at low concentrations does not allow the substrates to fully bond. Otherwise, a solvent excessively strong or at high concentration has the risk of microchannel clogging and distortion by excessively dissolving thermoplastic polymers. Trinh et al. introduced acetic acid as a solvent for clog-free bonding of PMMA microdevices at room temperature within 20 min [36]. Moreover, increased acetic acid concentration (10–100%) showed expansion of the bonding area; 50% acetic acid was the optimal concentration for completely bonding PMMA substrates [59]. In another study, UV exposure for 30 s and ethanol <50% showed reversible bonding, while ethanol >50% supported the irreversible bonding of PMMA assemblies [61]. Moreover, UV irradiation reinforces the activation of the thermoplastic surfaces in the presence of a solvent, thus, the monomers of two surfaces are rapidly activated and re-crosslinked to realize a permanent bond under relatively low pressure condition. For instance, 50% of acetic acid has been used to seal two PMMA substrates at room temperature for 20 min under a pressure of 0.4 MPa using a press machine [33]. UV-assisted acetic acid bonding required only 30 s of UV irradiation when assisted with clamps [59].

Effective and rapid thermoplastic bonding can be achieved by applying a mixture of different solvents. PMMA substrates were treated with acetone and ethanol (v:v, 8:2) for 30 s to fabricate a microfluidic chip without microchannel deformation [67]. COC chips were exposed to a mixture of 60% cyclohexane and 40% acetone (v:v) for 120 s to achieve high bond strength and good channel integrity [68]. Moreover, three critical components, i.e., acetone, n-pentane, and 1H,1H,2H,2H-perfluorooctyl trichlorosilane, assisted in one-step bonding of PC microfluidic chips within only 10 s [69]. One of the challenges of solvent bonding is the rapid evaporation of solvent near the free edges of microdevices due to the inherent volatility of solvent, which causes poor bonding and leakage. This phenomenon can be mitigated by adding grooves near the edges of microfluidic devices [61,70]. The addition of peripheral grooves is supported to retain the solvent, preventing evaporation during microwaving, and significantly improving the bonding coverage [71]. Further, the additional feature grooves substantially decreased the unbounded area surrounding individual microchannels [70]. A surface modification also suggests an improvement in solvent bonding. Ethanol and UV exposure of internal surfaces produces excellent bonding, increasing the bond strength between PMMA and acrylonitrile butadiene styrene [71]. In addition, surface chemical and plasma modification followed by solvent bonding suggest reproducible bonding in PMMA microfluidic devices [72,73].

### 3.3. Adhesive Bonding

**Figure 4:**
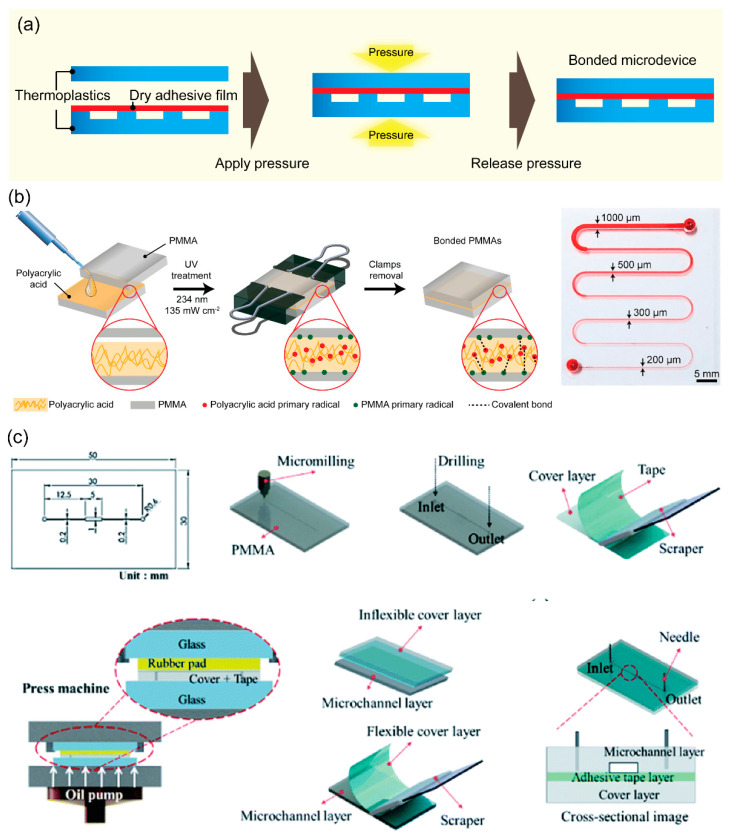
(**a**) Schematics showing the representative procedures for adhesive bonding using a dry adhesive film for fabricating thermoplastic microdevices. (**b**) Schematic illustration of the overall procedure for bonding PMMA device using PAA assisted by UV, the photograph of a clog-free PMMA microdevice including a serpentine microchannel with various channel dimensions. Adapted with permission from Ref. [77]. Copyright 2021, Elsevier. (**c**) Schematics showing the procedures of PMMA microdevice fabrication using an adhesive tape and press machine for bonding microdevice. Adapted with permission from Ref. [80]. Copyright 2020, Royal Society of Chemistry.

Adhesive bonding is a rapid and simple method for sealing thermoplastics where substrates are bonded at their interface by an adhesive (Figure 4c). Due to its simplicity, adhesive bonding is widely used for thermoplastics and for other materials. Liquid and dry adhesives can be used for specific requirements of thermoplastic bonding. A liquid adhesive usually requires a photo or thermal activation to form the bonded interface between two pieces of thermoplastic substrates. For example, microchannel and micropillar PMMA systems were bonded using a commercial UV adhesive (Slink 80801) with UV irradiation for 60 s [74]. Kratz et al. characterized four biomedical-grade pressure-sensitive adhesives (ARcare 92712, ARcare 90445, ARcare 90106, and ARseal 90880) for rapid prototyping of lab-on-a-chip systems; ARcare 90445 exhibited good bonding strength and gas tightness combined with satisfactory cell adhesion and viability [75]. In addition to commercial adhesives, several biopolymers can function as adhesion agents for bonding thermoplastic materials. Trinh et al. introduced the chitosan (CS)–polydopamine (pDA) hydrogel complex as an adhesion agent for reversible thermoplastic bonding assisted by UV irradiation [76]. Similarly, poly(acrylic acid) was adopted as UV-assisted adhesion promoter for fabricating thermoplastic microdevices [77]. A major challenge of adhesive bonding is channel clogging due to excessive liquid adhesive inside the microchannels. Therefore, several strategies such as adhesive printing, spin coating, and capillarity-driven adhesive delivery have been developed to prevent adhesive clogging [74,78].

Contrary to liquid adhesives, the simplest form of dry adhesive bonding is directly applying an adhesive tape onto thermoplastic substrates [79]. Tsao and Syu reported dry adhesive tape bonding of inflexible and flexible substrates using a manual scraper press and a hot press machine [80]. ORDYL dry film photoresist was used for packaging of COC microstructures; subsequently, oxygen plasma was used for adhesion improvement [81]. A thick adhesive film was applied for bonding multilayers of PMMA to form micropump with actuation chambers [82,83]. One advantage of adhesive bonding is the sealing of hybrid thermoplastic materials such as PMMA–PC, PMMA–PS, PMMA–PI, PMMA–PET, and PMMA–PVC [76,77] or a thermoplastic and elastomer (PMMA–PDMS) [83,84]. Song and Park used a 2.5% (w/w) PMMA solution as an adhesive layer to bond heterogeneous PMMA–PC polymers, by enclosing the PMMA microfluidic channels with PC [85]. Notably, the adhesive interface plays an important role in reversible bonding. Yao et al. reported a new reversible bonding strategy to seal conventional and hybrid reversible bonding (PMMA–PMMA or glass–PMMA) using UV release tape [86]. Thermoplastic bonding with a CS–pDA adhesion agent slightly decreased bond strength after four reversible bonding cycles [77].

### 3.4. Other Bonding Methods

In addition to the three major methods above, thermoplastic polymers can be sealed by other effective bonding methods such as physical-assisted bonding, chemical-assisted bonding, ultrasonic/laser welding, and microwave bonding. Physical modification of the surfaces can increase the surface energy, promoting bonding and enhancing the bonding strength between the two substrates. On the one hand, plasma and UV radiation are common physical agents for physical-assisted bonding. Plasma processing used deep O_2_ plasma etching on PMMA and a photosensitive PDMS as resist for the high-throughput mass production of polymeric microfluidic fabrication [87]. Vacuum UV (VUV) light irradiation, VUV irradiation in the presence of oxygen gas (VUV/O_3_), or O_2_ plasma treatment were used for direct bonding of two COP plates [88]. For example, Wen et al. employed a photo-bonding process with VUV light to fabricate microfluidic devices without using any solvent for cell culture applications [89]. To investigate the effects of residual solvent, the decrease in apoptosis was observed and compared with a device bonded using solvent. On the other hand, chemical-assisted bonding uses chemical reagents for the activation of thermoplastic surfaces for bonding. Surface modification-assisted bonding has been performed by plasma oxidation followed by tetraethyl orthosilicate treatment to facilitate siloxane bonding between the two polymer substrates (PMMA–PMMA and PMMA–PC) [90]. Surface modification of (3-aminopropyl)triethoxysilane has been shown to promote chemical bonding and robust irreversible bonding between PDMS and thermoplastics such as PS, PC, PMMA, and PET [91,92,93]. Moreover, Nguyen et al. reported a method for bonding PMMA to PET membranes using (3-glycidyloxypropyl)trimethoxysilane followed by air plasma and heating at 100 °C [94].

**Figure 5:**
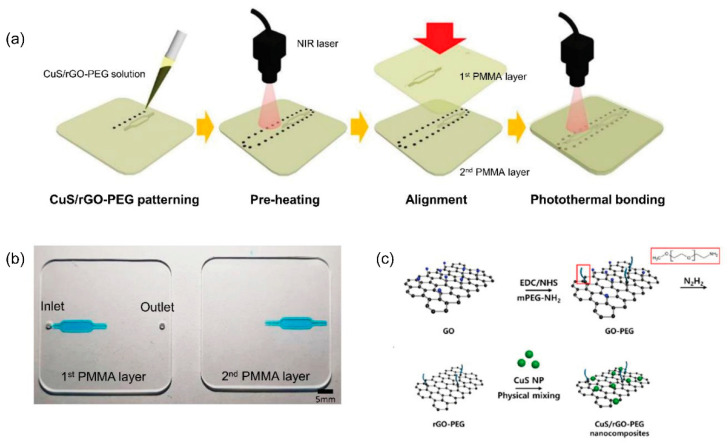
(**a**) Schematics showing PMMA bonding process using CuS/rGO-PEG nanocomposite and the photothermal effect. (**b**) Photographs of the PMMA device. (**c**) The synthesis process of the CuS/rGO-PEG nanocomposite [99].

Ultrasonic and laser welding accelerate bonding through local melting and welding. Ultrasonic bonding involves bonding through local melting by the propagation of ultrasonic sound; in contrast, laser bonding involves localized heating at the interface of two thermoplastic substrates [95,96]. Ultrasonic actuation has been applied for 10 s to preheated COC substrates to accelerate thermal compression bonding [97]. A diode laser has been used for micro-welding two PMMA substrates together using an intermediate thin film metal as a localized absorber [98]. Kim et al. introduced a new photothermal bonding of a PMMA device using copper sulfide/reduced graphene oxide-poly(ethylene glycol) (CuS/rGO-PEG) nanocomposites and near-infrared (NIR) laser irradiation (Figure 5) [99]. However, both ultrasonic and laser welding have limitations, including difficult adjustment of energy distribution and excessive fusion due to ultrasonic sound or laser pulses [100]. Alternatively, microwave bonding uses a microwave to heat the interface layer during bonding to produce bonding between thermoplastic substrates. Microwaving allows localized heating, which avoids excessive heat and prevents channel deformation. The thin film metal deposited on a PMMA substrate surface is designed to absorb microwave power, causing localized melting and improving adhesion at the interface for PMMA bonding [101]. Microwave bonding is a good alternative and user-friendly bonding method for thermoplastic microfluidic devices using a household microwave oven [59,101].

The typical bonding requirements, advantages, and disadvantages of several bonding methods are summarized in Table 1. Generally, several types of equipment such as a heater, press machine, ultrasonic/laser/microwave sources, and plasma/UV machine are required for thermoplastic bonding. With respect to reagents, these bonding methods require a variety of solvents, chemicals, and dry or liquid adhesives. Moreover, each approach has advantages and limitations.

## 4. Analytical and Diagnostic Applications

### 4.1. Nucleic Acid Diagnosis

**Figure 6:**
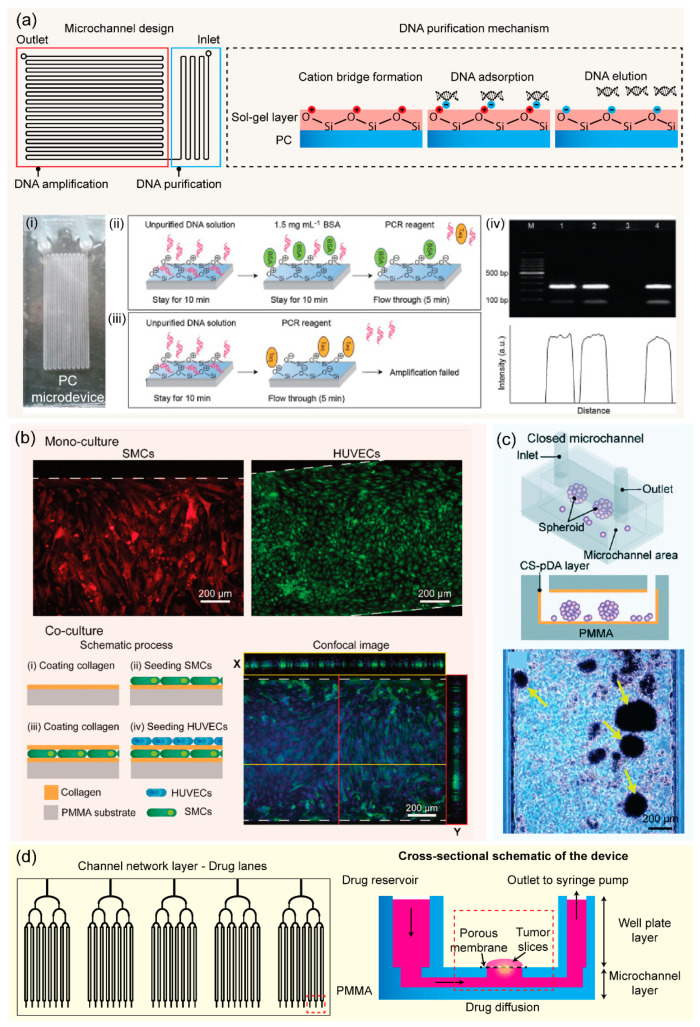
(**a**) Schemes illustrating a sol-gel coated polycarbonate (PC) microdevice for DNA purification and amplification, results of capturing of DNA using sol-gel coating layers used for DNA purification: (i) a photo of the purification microdevice, (ii,iii) schematic showing DNA elution using PCR reagent inside the microchannel with and without BSA treated, respectively, (iv) results of the PCR performed from the on-chip purification. Adapted with permission from Ref. [106]. Copyright 2014, Elsevier. (**b**) Results showing successful culture of SMCs and HUVECs inside a bonded PMMA microdevice using poly(acrylic acid) as an adhesion agent, schemes illustrating a layered co-culture model of SMCs and HUVECs using a poly(methyl methacrylate) (PMMA) microdevice. Adapted with permission from Ref. [77]. Copyright 2021, Elsevier. (**c**) Schematic representation of the MSC spheroids formed inside a closed-microchannel fabricated using the CS–pDA hydrogel complex, optical image showing MSC spheroids formed after five days of cell culture inside the microchannel, reproduced from [76]. (**d**) Schemes illustrating the PMMA platform for the drug-response testing system. Adapted with permission from Ref. [112]. Copyright 2020, Royal Society of Chemistry.

Nucleic acid (DNA and RNA) analysis is important for genetic research, disease diagnosis, and pathogen detection. PCR is one of the most robust nucleic acid amplification tools. Microfluidic PCR or CF-PCR permits rapid testing and identification of genetic samples with high throughput and high efficiency [103]. Since thermal bonding presents heat and chemical resistant ability, numerous PCR thermoplastic microchips have been developed for various applications [33,92,104]. A PS microdevice has been fabricated by micromilling replication and thermal bonding for pre-concentration and CF-PCR amplification of *E. coli* DNA [104]. Trinh et al. reported on an integrated monolithic PMMA microfluidic device for on-site detection of major foodborne pathogens in a continuous flow. The reported device consisted of a serpentine microchannel for on-chip amplification and a detection chamber for end-point fluorescence signal [105]. Zhang and co-workers presented a glass-like sol-gel (bis[3-(trimethoxysilyl)propyl]aminosilane) coating on the PC surface to facilitate one-step bonding of two PC substrates at a mild temperature under atmospheric pressure within 30 min [106]. In this case, sol-gel coated PC microchannel was employed for DNA purification, and integrated with a flow-through PCR to realize seamless DNA purification and amplification for rapid detection of *E. coli* using a monolithic PC device realized in 70 min (Figure 6a). Moreover, thermoplastic bonding is compatible with integrated surface plasmon resonance (SPR) fiber sensors. Solvent bonding exhibits a strong permanent bond of two substrates under mild pressure, allowing for the integration of sensor system into a microfluidic device. For instance, the integration of a microfluidic PCR device and SPR fiber sensor into one PMMA platform fabricated by ethanol solvent bonding was previously reported. This all-in-one system allowed DNA amplification-to-detection within 30 min through a digital SPR sensor signal [107]. Low pressure required in UV-assisted acetic acid bonding supported the integration of a platinum electrode array into a PMMA microfluidic device [59]. Another novel one-step method has great potential for manufacturing PC microfluidic chips for digital droplet PCR using ultrafast solvent bonding (acetone, n-pentane, and 1H,1H,2H,2H-perfluorooctyl trichlorosilane). Fortunately, 1H,1H,2H,2H-perfluorooctyl trichlorosilane plays a key role in the hydrophobic modification of the PC channel, significantly promoting the generation of monodisperse droplets [64]. In addition to PCR devices, thermoplastic microfluidics can apply novel isothermal amplification techniques for rapid and early pathogen detection [108,109]. Furthermore, adhesive bonding applies to fabricating point-of-care platforms since it is simple and low-cost, which meets the requirement of the point-of-care application. Centrifugal or foldable microdevices were fabricated by using thin PC and adhesive tape for multiple bacteria detections. These thermoplastic chips were integrated with DNA extraction, an isothermal amplification called loop-mediated isothermal amplification (LAMP), and colorimetric detections for multiplex point-of-care testing [110,111].

### 4.2. Cell-Based Analysis

Microfluidic platforms allow cell culturing and effective cell capturing, positioning, and analysis. Due to its biocompatibility, thermoplastic microfluidic devices have been widely applied for cell research. Several studies have reported various biocompatible and eco-friendly solvent bonding methods using acetic acid for PMMA microdevices. These bonded microdevices were successfully applied for culturing human cells such as human umbilical vein endothelial cells (HUVECs) and mesenchymal stromal cells (MSCs). This has provided good alternative platforms to perform on-chip viability assays [33,59,60]. Young et al. described the fabrication of PS microfluidic devices (hot embossing replication and thermal bonding) for two different cell-based applications including HUVECs activation and neutrophil chemotaxis [28]. Moreover, biopolymers have great potential as green materials for adhesive bonding of cell-based microfluidic devices due to their biocompatibility. Poly(acrylic acid) has been used as an adhesion promoter for UV-assisted bonding of thermoplastic microfluidic platforms in an in vitro blood vessel wall model. Smooth muscle cells (SMCs) and HUVECs have been cultured inside bonded microdevices in a co-culture model mimicking human blood vessels, applicable for organ-on-a-chip experiment (Figure 6b) [77]. A PMMA microdevice fabricated using the CS–pDA hydrogel complex and O_2_ plasma treatment promoted MSC proliferation and aggregation to form spheroids, allowing research on 3D human cell cultures (Figure 6c) [76]. In addition, several commercial adhesives, optically transparent and biocompatible, are available for fabricating microdevices for cell monolayers and 3D cell culture systems [75]. Thermoplastic devices are also useful for the validation of drug testing. PMMA–PET microfluidic devices sealed by chemical-assisted bonding were used to culture human lung adenocarcinoma cells. The PMMA devices exhibited more reliable cytotoxicity for vincristine (anticancer drug) as compared with conventional PDMS devices [94]. Rodriguez et al. developed a microfluidic platform for multiplexed drug testing of intact tumor slices from a patient’s colorectal tumor. The device was digitally manufactured in PMMA by CO_2_ laser micromachining and methylene chloride solvent bonding (Figure 6d) [112]. Thermoplastic devices have also been useful for on-chip electroporation of human cells to produce cell-free viruses. Using low-pressure solvent bonding, a PMMA microfluidic device was successfully integrated with microelectrode arrays, which continuously electrolyzed varicella-zoster virus-infected human foreskin fibroblasts for high-throughput production of cell-free viruses [113].

### 4.3. Other Analytical Applications

Protein and biomarker analyses are crucial in medical diagnostics and laboratory research. Due to the advantages of thermoplastics, ongoing efforts have focused on developing electrophoresis in microfluidic devices. Hot embossing and thermal bonding have been successfully applied to fabricate PMMA microfluidic systems for high-resolution electrophoretic separations of fluorescently labeled amino acids [42]. Similarly, a COC microfluidic device has been manufactured for reversed-phase electrochromatography separation of polycyclic aromatic hydrocarbons. These microstructures were fabricated by hot embossing and the microdevice was sealed by solvent-enhanced thermal bonding [36]. Wouters et al. reported on the use of COC microfluidic chips in high-performance liquid chromatography. The long straight separation channel layout was engraved by using a CNC micromilling robot, and solvent-vapor-assisted bonding was used to irreversibly seal the chips, producing the ideal channel geometry [114]. Moreover, an integrated PMMA microfluidic system has been fabricated to quantitatively determine fluorescently labeled α-fetoprotein (a biomarker for liver cancer) in human serum. The integrated microdevices were successfully applied for immunoaffinity purification, electrophoresis separation, and laser-induced fluorescence detection [115,116].

Table 2 comprehensively summarizes representative thermoplastic microfluidic systems for various applications. Numerous thermoplastic materials (PMMA, PS, PC, and COC) are commonly used for microfluidics. CNC micromilling is often applied for molding replications due to its automated and mass-producible properties apart from hot embossing and injection molding. Thermoplastic microfluidics are widely applicable to various fields such as integrated microfluidic systems, point-of-care devices, 2D/3D cell culture, organ-on-a-chip, drug testing, and microfluidic molecular separation/detection. A variety of bonding strategies such as thermal bonding, solvent bonding, and adhesive bonding are used for sealing microfluidic devices which are applied for various analytical and diagnostic applications. PCR microfluidic devices are operated in high temperature and high pressure conditions. For these reasons, thermal and solvent bonding are selected since the methods are highly resistant to high temperature and high pressure applications. Meanwhile, adhesive bonding using biocompatible materials such as hydrogels is suitable for fabricating cell-based microchips. Moreover, the modifications of the surface improve the functionality of the microfluidic channels including DNA purification, cell adhesion, or selective capture of biomolecules.

## 5. Conclusions and Future Perspective

In this review, we recapitulated the available knowledge of thermoplastic bonding for fabricating microfluidic devices as well as their applications. Numerous thermoplastic bonding approaches such as thermal bonding, solvent bonding, adhesive bonding, chemical/physical bonding, ultrasonic/laser welding, and microwave bonding are available for microfluidic devices. Researchers should select the appropriate bonding technique depending on the specific properties of the thermoplastic substrate and the requirements of the microfluidic chips. Furthermore, post-process applications could help determine the suitable approach for thermoplastic bonding. Typically, on the one hand, thermal bonding and solvent bonding techniques are used for thermal cycling (such as CF-PCR) microchips due to their thermostability and high bond strength. On the other hand, cell-based microdevices require biocompatible materials, preferring adhesive bonding.

With the development of microfluidic technology, thermoplastic microfluidics have great potential for applications such as nucleic acid analysis (DNA/RNA extraction, amplification, and detection), cell-based research (2D/3D cell culture, organ-on-a-chip, and drug-response testing), and electrophoresis. In addition to its robustness, low cost, and high throughput, its commercialization has allowed the research and development of thermoplastic microfluidic chips. In the future, microfluidic devices will become more complex and integrated, promoting all-in-one devices, wherein thermoplastic bonding allows large-scale bonding of multilayers and dissimilar materials.

## Figures and Tables

**Table 1:** Comparison of bonding methods for thermoplastic devices.

Bonding Method	Bonding Requirement	Advantages	Disadvantages	Ref.
Thermal bonding	Heat and pressure Hot press machines	Simplicity and robustness Thermostability	Risk of microchannel deformation Requirement of bulky press machine or heater	[43,47,49]
Solvent bonding	Solvent solutions Press machines or clamps	Rapid and low cost Mild pressure Strong bond strength	Risk of microchannel clogging Risk of solvent volatility and flammability	[56,61,63]
Adhesive bonding	Dry or liquid adhesives Press devices or surface treatment	Hybrid materials bonding Simplicity and low cost Biocompatibility	Risk of microchannel clogging Limited heat resistance	[76,81,83,84]
Physical-assisted bonding	Plasma treatment UV radiation	Simplicity and straightforward Mild conditions	Requirement of bulky equipment Relatively low bond strength	[87,88]
Chemical-assisted bonding	Chemical reagents Surface treatment	Robustness Thermostability Hybrid materials bonding	Chemical toxicity Time-consuming surface modification Multi-step coating process	[90,91,92,93,94]
Ultrasonic/laser welding	Ultrasonic sound Laser pulse	Robustness and straightforward Thermostability	Difficulty in adjusting energy distribution Risk of excessive fusion	[97,98,99,100]
Microwave bonding	Microwave sources Surface treatment	Rapid and low cost User-friendly process	Requirement of optimization Requirement of additional treatment	[101,102]

**Table 2:** Representative thermoplastic bonding strategies and applications.

Application	Molding Replication	Bonding Method	Surface Treatment	Significant Results	Ref.
Microfluidic PCR	PS CNC micromilling	Thermal bonding (100 °C, 0.1 MPa, 10 min)	No treatment	Pre-concentration of bacteria CF-PCR amplification	[104]
	PMMA CNC micromilling	Thermal bonding (105 °C, 0.1 MPa, 30 min)	No treatment	DNA purification Flow-through PCR amplification Fluorescence detection	[105]
	PC CNC micromilling	Thermal bonding (128 °C, 0.1 MPa, 30 min)	Bis[3-(trimethoxysilyl)propyl]aminosilane coating	DNA purification Flow-through PCR amplification	[106]
PCR–Surface plasmon resonance (SPR) device	PMMA CNC micromilling	Solvent bonding (90% ethanol, UV light)	No treatment	Integration of SPR fiber Flow-through PCR amplification Digital SPR sensor signal detection	[107]
Droplet microfluidic chip	PC Injection molding	Solvent bonding (Acetone and n-pentane)	Hydrophobic surface modification by 1H,1H,2H,2H-perfluorooctyl trichlorosilane	Generation of monodisperse droplets Digital droplet PCR Microscopic fluorescence detection	[69]
Point-of-care devices	Thin PC CNC micromilling	Adhesive bonding (Adhesive tape)	No treatment	DNA extraction LAMP amplification Naked eye detection	[110,111]
Cell culture	PMMA CNC micromilling	Solvent bonding (50% acetic acid)	Improving cell adhesion by fibronectin treatment	2D HUVECs culture On-chip viability assay analysis	[33]
	PMMA CNC micromilling	UV-assisted solvent bonding (50% acetic acid)	No treatment	2D MSCs culture On-chip viability assay analysis	[59]
	PMMA CNC micromilling	Microwave-assisted solvent bonding (60% acetic acid)	No treatment	2D cell culture (HUVECs and MSCs) On-chip viability assay analysis	[60]
	PS Hot embossing	Thermal bonding (90 °C, 13.2 kPa, 30 min)	Improving cell adhesion by O_2_ plasma treatment	HUVECs culture Neutrophil chemotaxis	[28]
Organ-on-a-chip	Thermoplastics CNC micromilling	Adhesive bonding (CS–pDA, UV irradiation)	O_2_ plasma treatment	3D cell culture MSCs spheroids formation	[76]
	Thermoplastics CNC micromilling	UV-assisted adhesive bonding (Poly(acrylic acid)	Collagen coating	SMCs and HUVECs co-culture In vitro model of human blood vessels	[77]
Drug-response testing	PMMA–PETE CNC micromilling	Chemical-assisted bonding (Air plasma and (3-glycidyloxypropyl)trimethoxysilane)	Fibronectin and collagen treatment	Culture of human lung adenocarcinoma cells Cytotoxicity for vincristine (anticancer drug)	[94]
	PMMA CO_2_ laser micromachining	Solvent bonding (Chloroform and methylene chloride)	Chloroform treatment	Culture intact tumor slices Multiplexed drug testing	[112]
Microfluidic cell lysis	PMMA CNC micromilling	Solvent bonding (acetic acid, 0.4 MPa, 30 min)	Bovine serum albumin (2%) treatment	Integration of arrays of microelectrode High-throughput production of cell-free viruses	[113]
Microfluidic separation	COC Robotic micromilling	Solvent-vapor-assisted bonding (Cyclohexane, 15 min)	Methanol treatment	Integration of the stationary phase High-performance liquid chromatography	[114]
Biomarker detection	PMMA Hot embossing	Thermal bonding (110 °C)	Immobilization of anti-α-fetoprotein	Microfluidic immunoaffinity extraction Quantification of α-fetoprotein	[116]

## Data Availability

Not applicable.
